# Induction of *lcc2* expression and activity by Agaricus bisporus provides defence against Trichoderma aggressivum toxic extracts

**DOI:** 10.1111/1751-7915.12277

**Published:** 2015-03-30

**Authors:** Calvin P Sjaarda, Kamal S Abubaker, Alan J Castle

**Affiliations:** Department of Biological Sciences, Brock UniversitySt Catharines, ON, L2S 3A1, Canada

## Abstract

Laccases are used by fungi for several functions including defence responses to stresses associated with attack by other fungi. Laccase activity changes and the induction of two laccase genes, *lcc*1 and *lcc*2, in *Agaricus bisporus* were measured in response to toxic extracts of medium in which *Trichoderma aggressivum*, the cause of green mould disease, was grown. A strain of *A. bisporus* that shows resistance to the extracts showed higher basal levels and greater enzymatic activity after extract exposure than did a sensitive strain. Furthermore, pre-incubation of *T. aggressivum* extract with laccases reduced toxicity. Faster induction and greater numbers of *lcc*2 transcripts in response to the extract were noted in the resistant strain than in the sensitive strain. The timing and increase in *lcc2* transcript abundance mirrored changes in total laccase activity. No correlation between resistance and *lcc*1 transcription was apparent. Transcript abundance in transformants with a siRNA construct homologous to both genes varied widely. A strong negative correlation between transcript abundance and sensitivity of the transformant to toxic extract was observed in plate assays. These results indicated that laccase activity and in particular that encoded by *lcc*2 contributes to toxin metabolism and by extension green mould disease resistance.

## Introduction

Interactions between fungal species are extremely common in natural ecosystems where species compete for limited resources and space for reproduction. Competition and antagonism are frequently manifested by mycoparasitism (Chet *et al*., 1981), the production of toxins (Reino *et al*., 2008) and/or cell wall degrading enzymes (Yang *et al*., 2009) that impair growth of a neighbouring species. Both toxin and cell wall degrading enzymes are observed during confrontation between *Trichoderma aggressivum* and *Agaricus bisporus*, the button mushroom (Krupke *et al*., 2003; Guthrie and Castle, 2006; Abubaker *et al*., 2013).

In the mid-1980s, extensive crop losses attributed to *T. aggressivum* f. *europaeum* occurred on mushroom farms in the British Isles (Seaby, 1987). Over time, the disease spread across Europe and into Western Asia (Geels, 1997; Mamoun *et al*., 2000; Sobieralski *et al*., 2009). Similar problems developed in Ontario and British Columbia in the early 1990s. The causative agent, *T. aggressivum* f. *aggressivum*, has subsequently caused severe losses in many areas across North America (Rinker, 1993; Rinker and Alm, 1997; Anderson *et al*., 2001). This problem is known worldwide as ‘green mould disease’. The very common off-white strain U1, its derivatives and white strains are very sensitive to *T. aggressivum*. Commercial brown strains are much more resistant, and crop loss is much less severe than that of the U1 strain (Rinker and Alm, 1997; Anderson *et al*., 2001). Green mould disease is currently controlled by careful application of fungicides such as the benzimidazole-based types that inhibit *T. aggressivum* but not *A. bisporus*, by strict sanitation procedures on mushroom farms and in some countries, by treatment with the biocontrol agent, Serenade (Largeteau and Savoie, 2010). Future control, however, could come from procedures that are based on an understanding of the mechanisms of interaction between the two fungal species. Approaches may be to understand and subsequently alter the response of *A. bisporus* to *T. aggressivum*, or develop markers to select strains with increased resistance to *T. aggressivum*.

*Trichoderma aggressivum* produces 3,4-dihydro-8-hydroxy-3-methyl isocoumarin, a toxin that inhibits the growth of *A. bisporus* (Krupke *et al*., 2003). The inhibition of a brown strain, SB65, was significantly less than that observed with the white strain S130, an observation that is consistent with commercial brown strain resistance to green mould disease. The mechanism of resistance is unknown but one plausible explanation is that commercial brown strains are able to degrade the toxin more rapidly than white or off-white strains. If so, possible candidate enzymes would include laccases.

Laccases (benzenediol : oxygen oxidoreductase, EC 1.10.3.2) are extracellular, multicopper enzymes that use molecular oxygen to degrade numerous phenolic substrates. The main role of these enzymes in basidiomycetes is lignin utilization (Leonowicz *et al*., 2001), but reports indicate a wide range of functions including defence against stressful conditions (Thurston, 1994; Mayer and Staples, 2002; Baldrian, 2004), melanin synthesis (Nagai *et al*., 2003) and breakdown of toxic xenobiotic, phenolic compounds and secondary metabolites (Mayer and Staples, 2002; Baldrian, 2006). Several fungal antagonists, including *Trichoderma harzianum*, have been shown to increase laccase activity in the basidiomycetes *Trametes versicolor* and *Pleurotus ostreatus* during co-cultivation (Baldrian, 2004). Laccase gene transcription and isozyme abundance are increased in *P. ostreatus* in response to *Trichoderma longibrachiatum* (Velázquez-Cedeño *et al*., 2007). When challenged with *T. harzianum* mycelium or spent medium from *T. harzianum* culture, *Lentinula edodes* responds with an increase in laccase activity (Savoie *et al*., 1998; 2001). The resistance of *L. edodes* to its own variety of green mould disease can be improved by alteration of environmental conditions with the addition of lignin and phenolic compounds to the substrate to stimulate laccase production (Savoie and Mata, 2003). Similar interventions for decreasing damage by *T. aggressivum* during button mushroom production may be possible.

This work summarizes an initial examination of the roles of laccases in the response of *A. bisporus* to *T. aggressivum* toxin. The specific objectives were to determine if *T. aggressivum* toxin was inactivated by laccase, if toxic extracts induced laccase enzymatic activity and transcription in *A. bisporus*, and if induction was more pronounced in a green mould-resistant commercial brown strain than in a sensitive off-white strain. Growth responses of *A. bisporus c*ultures were assessed following exposure to toxic extracts of *T. aggressivum* prior to and after laccase treatment. Combined activities of all laccases and transcript abundances of two laccase genes, *lcc*1 and *lcc*2, in response to the extract were measured. The upstream regulatory elements of *lcc*1 and *lcc*2 were also sequenced from both brown and off-white strains to see if any dissimilarity in transcriptional control sequences could account for any differences in transcript abundance between the strains. Finally, siRNA constructs were introduced into both strains and were used to correlate *lcc*1 and *lcc*2 transcript abundance with sensitivity of *A. bisporus* to *T. aggressivum* toxic extracts.

## Results

### Toxicity of *T. aggressivum* secondary metabolites

**Figure 1:**
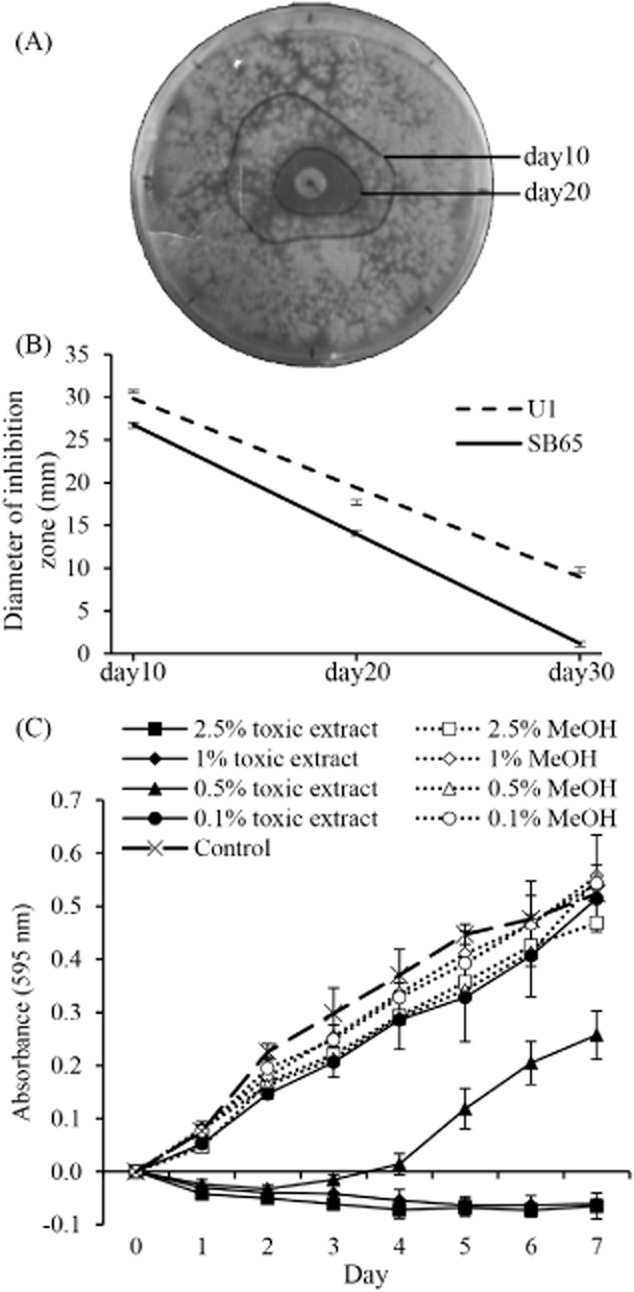
Inhibition of *Agaricus bisporus* growth by *Trichoderma aggressivum* toxic extract.A. With time, *Agaricus* colonized the zone of inhibition produced by *T. aggressivum* extract. The outer line demarcates the zone of inhibition after 10 days and the inner line after 20 days incubation with a paper disc impregnated with extract.B. The zone of inhibition was smaller for SB65 than for U1 at all three measurements and SB65 was able to overcome the toxicity faster than U1 (*n* = 10).C. In a microtiter plate assay, extract concentration at 0.1% v/v had no effect on growth, at 0.5% *Agaricus* was initially inhibited by the extract but was able to overcome toxicity, and at > 1%, *Agaricus* did not grow (*n* = 4).

Toxicity of *T. aggressivum* extract to *A. bisporus* was measured as a zone of inhibition surrounding the extract impregnated paper disc every tenth day for 30 days (Fig. 1A). The extract resulted in a significantly larger inhibition zone (*P* < 0.001) with U1 than SB65. With time, the zones diminished (Fig. 1A and B) indicating that the toxicity had decreased significantly (*P* < 0.001), allowing both strains of *A. bisporus* to colonize the inhibition zone. Colonization of the zone was visualized as the appearance of discrete colonies rather than as continued growth from the edge of the non-inhibited mycelium. These observations suggested that the toxic effect is fungistatic rather than fungicidal. The rates at which the two *A. bisporus* strains grew within the inhibition zone were unequal, with the brown strain, SB65, colonizing faster than the U1 off-white strain (Fig. 1B; *P* = 0.001). The differences in the rate of colonization suggested that the reduction in toxicity with time was not due solely to evaporation or abiotic degradation of the toxin.

When *T. aggressivum* extract (0.5% v/v) was used in liquid culture, the growth of *A. bisporus* U1 was inhibited for the first 4 days (Fig. 1C). However, by day 5, *A. bisporus* was able to overcome the toxicity and growth was apparent as a significant increase in absorbance (*P* < 0.001). Concentrations of 1% or 2.5% of *T. aggressivum* extract in the medium completely inhibited growth of *A. bisporus* through the entire 7 days of the experiment (*P* < 0.001). At 0.1% final concentration, no inhibition of growth was observed.

### Detoxification using exogenous laccase

**Figure 2:**
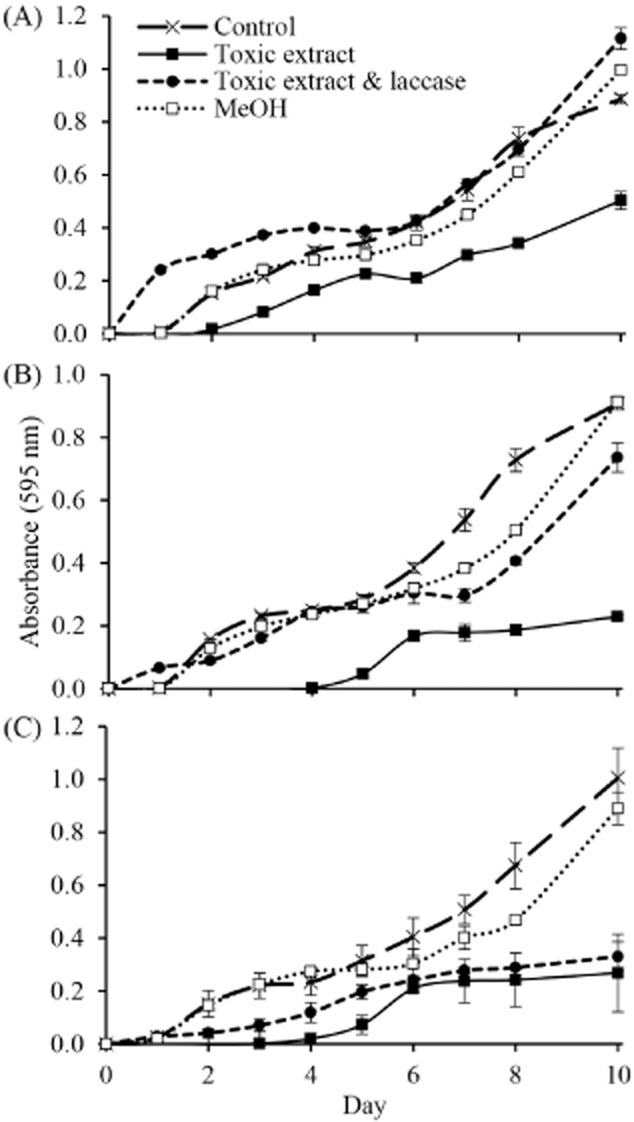
Reduction of *T. aggressivum* extract toxicity by treatment with *A. bisporus* laccase. Extracts (A) 0.25%, (B) 0.5% and (C) 0.75% v/v were incubated overnight with exogenous laccase before growth tests with *Agaricus* U1 cultures (*n* = 4). At the two lowest concentrations (A and B), extract toxicity was reduced by laccase allowing for *Agaricus* growth similar to control tests with buffer or methanol (MeOH).

Based upon the results in Figure 1C, *T. aggressivum* extract in concentrations of 0.25%, 0.5% and 0.75% v/v were used to test the effect of *A. bisporus* laccases on detoxification (Fig. 2). Each concentration inhibited the growth of *A. bisporus* compared with water or methanol controls. At each concentration, *A. bisporus* was able to overcome the toxic effect and resume growth after 3 days (0.25%) or 5 days (0.5% and 0.75%). When laccase was added to the extract prior to incubation with *Agaricus*, the toxicity was reduced in both the 0.25% and 0.5% tests, and *A. bisporus* showed growth rates similar to the controls. Laccase activity had no significant effect on the growth of *A. bisporus* at a final extract concentration of 0.75%.

### Measurement of total laccase activity

**Figure 3:**
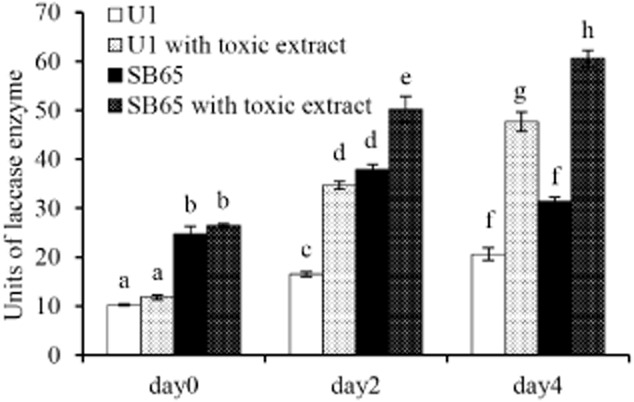
Induction of laccase activity by *T. aggressivum* extract. *Agaricus bisporus* laccase enzyme activity was measured over 4 days in culture with and without the presence of *T. aggressivum* extracts. Letters designate statistical differences within each day [ANOVA – Bonferroni post-hoc (*P* ≤ 0.05)]. Each bar represents the mean (± standard error (SE)) of three replicates.

If laccases do have a role in green mould disease resistance, then two reasonable predictions are that a resistant strain of *A. bisporus* should produce more laccase than a sensitive one and that activities should increase upon exposure to the toxin. Laccase activity at day 0 was twofold higher in resistant SB65 than sensitive U1 in the control, untreated cultures (Fig. 3, *P* = 0.001). Over the 4-day incubation, activity increased slightly in the U1 control, and by day 4, there was no significant difference between the two strains (*P* = 0.078). The addition of the *T. aggressivum* extract caused a significant increase in laccase activity in both strains of *A. bisporus* (*P* < 0.001). At days 2 and 4, both strains displayed increased activity in the presence of extract, yet the activity was always higher in the SB65 strain than in U1 (*P* ≤ 0.05).

### Measurement of *lcc*1 and *lcc*2 transcript abundance

**Figure 4:**
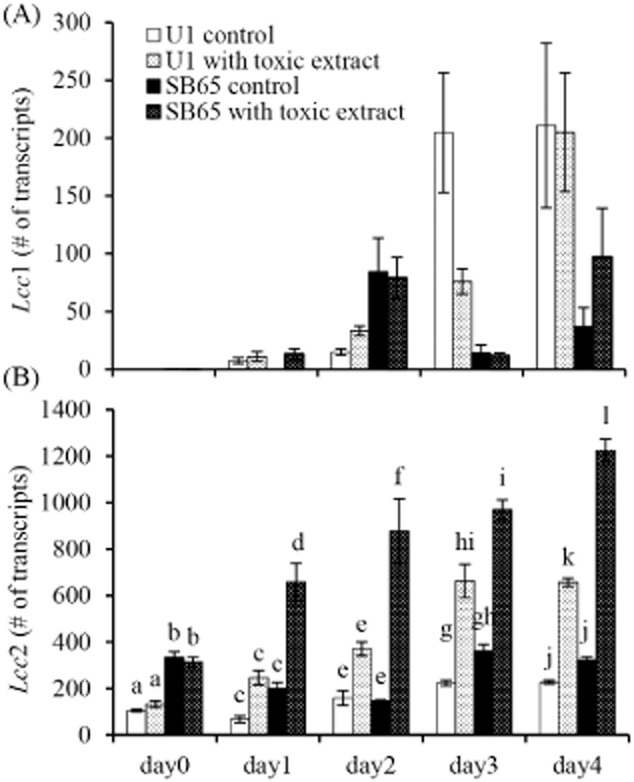
Induction of *lcc*2 transcription by *T. aggressivum* extract.A. *Agaricus bisporus lcc*1 transcript abundances were measured over 4 days in culture with and without the presence of *T. aggressivum* extract.B. *Agaricus bisporus lcc*2 transcript abundances were measured over 4 days in culture with and without the presence of *T. aggressivum* extract. Significant differences on each day are indicated by different letters [ANOVA – Bonferroni post-hoc (*P* < 0.05)]. Each bar represents the mean (± standard error (SE)) of three replicates.

In order to examine the transcriptional responses of selected laccase genes, *lcc*1 and *lcc*2 transcript abundances were measured by quantitative reverse transcriptase polymerase chain reaction (qRT-PCR) in *A. bisporus* strains that had been challenged with the extract. At the onset of the experiment, *lcc*1 was not expressed in either the off-white or brown strains (Fig. 4A). By day 4, *lcc*1 transcription had increased significantly (*P* = 0.002) in both strains. However, there was no difference in transcript abundance between U1 and SB65 throughout the test (*P* = 0.408), nor did the presence of *T. aggressivum* extract affect transcript abundance, with insignificant differences between the treated and untreated samples (*P* = 0.117).

No significant increase in *lcc*2 transcript abundance was observed in the untreated control cultures over time (Fig. 4B). However, in extract-treated samples, both strains of *A. bisporus* showed significant increases in the number of *lcc*2 transcripts over the 4-day period (*P* < 0.001). In addition, the number of transcripts was significantly greater in extract-treated SB65 than in U1 for all days (*P* < 0.001).

One-way analysis of variance (ANOVA) showed that there was a statistically significant difference between the groups at all five time points (day 0, *P* = 0.001; day 1, *P* = 0.004; day 2, *P* = 0.008; day 3, *P* = 0.001; day 4, *P* < 0.001). At day 0, the brown strain SB65 produced threefold more *lcc*2 than the off-white strain U1. In the presence of extract, the number of *lcc*2 transcripts increased significantly by day 1 in SB65 and by day 3 in U1. The number of transcripts in SB65 had increased to over 1200 transcripts per 1000 copies of *β-tub* transcript by day 4, threefold higher than the control and almost double the number of U1 transcripts in the same conditions.

### Regulatory elements of *lcc*1 and *lcc*2

In an attempt to explain the differences in gene expression between the two *A. bisporus* strains, we compared the regulatory regions of both genes from both strains. The *lcc*1 regulatory region (−422 to 0 bp) was 100% homologous in both U1 and SB65. Immediately following *lcc*1 and up to −460 bp of the *lcc*2 start codon lies a spacer of 1026 bp. This spacer was different at 17 nucleotides between the two strains. The differences did not appear to affect any presumptive regulatory motifs. The *lcc*2 regulatory region (−460 to 0 bp) was 99.8% homologous in both strains, differing in only one nucleotide at position −334. The polymorphism results in a TATA box in SB65 but this change is most likely neutral because the position is too far upstream to affect transcription, and the TATA box activity as predicted by its structure, 5′ TATACTA 3′, is likely too weak at 3% (Juo *et al*., 1996) to have any significance effect on transcript abundance.

Differences in transcript abundances of *lcc*1 and *lcc*2 under various environmental conditions have been noted previously (Smith *et al*., 1998; Morin *et al*., 2012), although explanations for the regulatory mechanisms that result in the differences are lacking. The promoters of *lcc*1 and *lcc*2 were examined for any regulatory elements that might account for this variation. Both genes had the same general transcription factor elements, TATA and CCAAT boxes, in the same positions described by Smith and colleagues (1998). Several specific regulatory elements were also found in both promoters. These included CreA (cAMP-mediated glucose repression) sites and several nitrogen repression response element (Nit2) sites. Allowing for one mismatch from the TGCRCNC consensus sequence (Eastwood *et al*., 2011), six putative metal response elements were found for *lcc*1 and two preceded *lcc*2. A humic regulatory response element (Morin *et al*., 2012) was detected in promoters of both genes but *lcc*1 appeared to be heteroallelic and *lcc*2 homoallelic in both U1 and SB65 (Table 1). The heteroallelic state for *lcc*1 in strain U1 was inferred from the sequences recovered in the present work, which were identical to the information in Perry and colleagues (1993), and from the sequence given in Morin and colleagues (2012) on an alternate allele in the H97 homokaryon derived from U1. Some regulatory sites were observed in *lcc*1 but not *lcc*2. A heat shock element [likely to be one with a low affinity for a heat shock factor because it has the required nGAAn motif (Sorger, 1991) but has only two complementary repeats, both with mismatches] occurred in the *lcc*1 promoter but not *lcc*2. The *activation of cup1 expression* (ACE1) response element, often present in the promoter regions of some multicopper oxidase genes (Janusz *et al*., 2013), occurred in the reverse orientation just 17 bp upstream from the translation start codon of *lcc*1 but was not found in the *lcc*2 promoter. Similarly, a xenobiotic response element (Janusz *et al*., 2013) was found in the *lcc*1 promoter but not in *lcc*2. One regulatory element was associated with *lcc*2 but not *lcc*1; two copies of the general stress response element (STRE; 5′ CCCCT 3′; Kobayashi and McEntee, 1993) were found upstream of *lcc*2.

**Table 1:** Humic response elements in *lcc*1 and *lcc*2

Gene	Sequence	Comments	Reference
	TCMKGWDAMAATCTC	HRE Consensus	Morin *et al*. (2012)
*lcc*1	TCCTGAAAAA-TTTT	U1 homokaryon H97	Morin *et al*. (2012)
*lcc*1	T–TTAGAAAATTTC	D649	Smith *et al*. (1998)
*lcc*1	T–TTAGAAAATTTC	U1	Present study
*lcc*1	TCCTGAAAA–TTTT	SB65 (allele 1)	Present study
*lcc*1	T–TTAGAAAATTTC	SB65 (allele 2)	Present study
*lcc*2	TCCTGAAAAAATTTC	U1 homokaryon H97; D649; U1; SB65	Morin *et al*. (2012); Smith *et al*. (1998); Present study

### siRNA and *lcc*1 and *lcc*2 expression

*Agrobacterium tumefaciens*-mediated transformation was attempted with vegetative hyphae of both strains of *A. bisporus*. Numerous trials using this tissue failed to yield any transformants. Therefore, off-white and brown mushrooms with unopened caps were purchased from a local grocery store for transformation trials with gill tissue according to Chen and colleagues (2000). Transformation using gill tissue was successful in 12.5% of off-white mushroom slices and 7.5% of brown mushroom slices. Four transformants from the off-white mushrooms and three from the brown mushrooms were recovered. The presence of the siRNA cassette in all isolates was confirmed by PCR.

**Figure 5:**
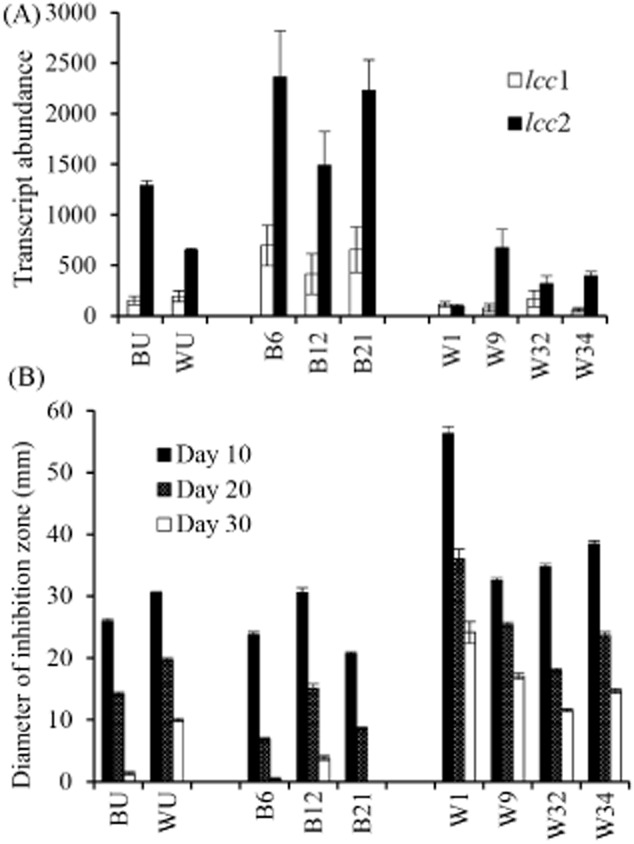
Effect of an siRNA construct on *lcc*1 and *lcc*2 transcription and growth sensitivity to *T. aggressivum* extract.A. Number of *lcc*1 and *lcc*2 transcripts measured by quantitative reverse transcriptase polymerase chain reaction (qRT-PCR) in the brown and off-white strains of mushroom containing the construct for RNA silencing in liquid culture containing *T. aggressivum* extract (*n* = 3).B. Inhibition zones resulting from the presence of toxic extract from *T. aggressivum* of *A. bisporus* off-white and brown strains containing the siRNA construct (*n* = 10). BU, WU, untransformed brown and off-white controls; B#, W#, transformed brown and off-white isolates, different numbers indicate independently isolated transformants.

The siRNA construct had varying effects on laccase RNA transcript levels as measured after a 4-day challenge with the *T. aggressivum* extract (Fig. 5A). Two of the brown transformants (B6 and B21) showed significant increases in transcription of both laccase genes, and transformant B12 was similar to the untransformed brown control. Transcript abundance in the untransformed brown control was virtually identical to SB65 treated with the extract for 4 days (Fig. 4A and B). In the off-white background, transformant W1 displayed efficient knockdown of both *lcc*1 and *lcc*2, W32 and W34 had slightly reduced *lcc*2 expression, and W9 showed no difference from the untransformed off-white control. Transcript abundance in the untransformed off-white control was the same conditions as in U1 treated for 4 days (Fig. 4A and B).

The level of laccase transcription had an inverse correlation with the susceptibility of the transformed *A. bisporus* to *T. aggressivum* extract (Fig. 5A and B). Transformants that overexpressed the laccase genes (B6 and B21) were more resistant than the untransformed control, while a transformant with a very low number of laccase transcripts (W1) was very sensitive. Transformants with laccase transcription levels similar to the controls also had toxic extract sensitivity that was similar to the controls.

## Discussion

Many fungi increase their fitness by producing toxins that inhibit the growth of other fungal, bacterial, plant or animal species. Toxin production can occur during parasitism of a target species, competition for resources or as a protective mechanism during the transition from vegetative growth to sporulation. In response to contact with toxins, resistance may be achieved by inhibition of further production. In an interaction with *Fusarium graminearum*, *T. harzianum* produces 6-pentyl-α-pyrone (6-PPO) that inhibits synthesis of the toxin deoxynivanol by the former species (Cooney *et al*., 2001). Resistance can also be conferred by metabolism of the toxin by the interacting species. For example, the pentyl moiety of 6-PPO is hydroxylated and further oxidized by *Botrytinia cinerea*, *Sclerotinia sclerotiorum* and several *Fusarium* species (Poole and Whitaker, 1997; Cooney and Lauren, 1999), changes that reduce hydrophobicity and toxicity. The enzymes that catalyse these reactions, however, remain unidentified.

The diffusion assay results in the present study provided evidence for a role of enzymatic activities of *A. bisporus* in resistance to green mould disease. The U1 strain was significantly more sensitive to the toxic components of *T. aggressivum* organic extracellular extracts than SB65 (Fig. 1) confirming the results of Krupke and colleagues (2003). With time, both strains were able to grow over the inhibition zone. This recovery may have resulted from toxin volatility or degradation through abiotic or enzymatic mechanisms. Loss of toxicity due to volatility and abiotic breakdown would have occurred at a similar rate in both strains. SB65, however, was able to overcome inhibition at a significantly faster rate than U1 suggesting the involvement of a strain-specific factor, possibly an enzyme, in the recovery. Given the structure of the toxin, a methylisocoumarin, proteins with phenolic oxidizing activities such as laccase and manganese peroxidase are likely candidates for the pertinent enzyme. Transcript abundance of the *A. bisporus* manganese peroxidase gene did not change significantly in response to *T. aggressivum* extract and did not correlate with resistance to green mould disease (data not shown) indicating that this enzyme is likely not directly involved with toxin catabolism. Indirect evidence suggests that laccase activity may serve a role in resistance to fungal toxins. Laccase expression is induced in numerous fungi by a wide variety of phenolic compounds (Piscitelli *et al*., 2011), likely including several toxins. *Trichoderma harzianum*, which secretes 6-PPO, stimulates laccase activity in *Serpula lacrymans* (Score *et al*., 1997) and *L. edodes* (Savoie *et al*., 1998; 2001). *Lentinula edodes* treated with extracellular metabolites of *T. harzianum* also showed increased laccase production (Savoie *et al*., 1998). Savoie and colleagues (2000) showed that amendment of the growth medium of *L. edodes* with lignin and phenolic compound-rich substrates reduced the competitive abilities of *Trichoderma* spp. and suggested that laccase induction could have contributed to this decrease.

This report provides direct evidence for a role of laccases in resistance to *T. aggressivum* toxin. When the *Trichoderma* extract was incubated overnight with *A. bisporus* laccases, the toxicity of the extract was significantly reduced, allowing *Agaricus* to grow similarly to the no toxin controls (Fig. 2). No reduction of toxicity by laccase treatment was observed at the highest extract concentration. This observation can be explained by the presence of an inhibitor of laccase activity in the extract or by an overabundance of toxin. Neither explanation can be discounted based upon current observations. Further evidence for a role of laccases in *Agaricus*’ defence against green mould diseases was provided by the comparison of laccase activities in toxin resistant (SB65) and sensitive (U1) strains of *A. bisporus*. Basal activity was significantly higher in SB65 than U1 (Fig. 3). In addition, laccase activity was inducible in both U1 and SB65 strains by the presence of *T. aggressivum* extract, yet SB65 always showed higher laccase activity than U1. Laccase substrates have been difficult to define because the enzymes lack specificity and their activity spectrum overlaps with those of other oxidases (Thurston, 1994). Their broad substrate range includes polyphenols, methoxy-substituted phenols, aromatic diamines and others (Trejo-Hernandez *et al*., 2001; Baldrian, 2006). Laccases have at least one type I (T1) copper centre that removes one electron from an aromatic carbon to generate a free radical (Thurston, 1994; Larrondo *et al*., 2003). The free radical is unstable and can be degraded by enzymatic or non-enzymatic reactions. Laccases can enzymatically remove a second electron to generate a quinone or the radicals can undergo non-enzymatic hydration or polymerization (Thurston, 1994). It is possible that radicals generated by laccases non-specifically polymerize the methylisocoumarin toxin produced by *T. aggressivum* thereby reducing toxicity.

If laccases were to be involved in alteration of the *T. aggressivum* toxin, a reasonable expectation is that the genes should either be constitutive with relatively high transcript abundances in the absence of the toxin or show significant induction following toxin exposure. Based upon this expectation, *lcc*1 does not appear to be involved in toxin resistance. Initially, *lcc*1 expression was very low, and subsequent days showed control cultures with similar or greater expression than cultures treated with the toxin (Fig. 4A). The low basal expression was consistent with the observations of Smith and colleagues (1998) who described 7000-fold greater *lcc*2 expression than *lcc*1 on malt extract medium. In contrast, Morin and colleagues (2012) measured roughly equivalent expression of the two genes on a defined minimal medium. Minimal medium contains significantly lower concentrations of carbon and nitrogen sources than the medium used by Smith and colleagues (1998) or in this study. The *lcc*1 promoter contains numerous CreA and nit2 elements that likely mediated the variable expression observed in the three studies and possibly the increase in transcription seen over time in the present work as nutrients were depleted. Overall, regulation of *lcc*1 expression in these tests is better explained by nutritional demands than any response to the toxin. Several observations indicated that laccase 2 serves a role in toxin resistance: (i) transcript abundance of *lcc*2 was consistently higher than *lcc*1 (Fig. 4A and B); (ii) there was a significantly greater transcript abundance of *lcc*2 in the resistant SB65 strain than in sensitive U1 throughout the study and, most notably, prior to toxin exposure; (iii) the addition of toxin had a significant inductive effect in both strains; and (iv) resistant SB65 responded quickly to the presence of toxin and *lcc*2 transcript abundance increased significantly within 24 h whereas in sensitive U1 the increase was delayed until day 3 (Fig. 2B).

Explanations for the differences in expression of *lcc*1 and *lcc*2 are not readily apparent, but examination of the promoters does provide some insight. Repression of *lcc*1 but not *lcc*2 is unlikely because the only site of repressor binding that was detected, the CreA element, was present in multiple copies in both promoters. The most likely candidates for differential gene induction would either be the general stress response element, STRE (Kobayashi and McEntee, 1993), or the humic response element (HRE) (Morin *et al*., 2012). Two STRE were found in the promoter of *lcc*2 and none in *lcc*1. STRE mediates response to nitrogen starvation, heat shock, osmotic shock and a variety of chemical stressors (Treger *et al*., 1998). It is possible that this element also controls response to the *T. aggressivum* toxin. The HRE was found in the promoters of both genes but was heteroallelic in *lcc*1 and homoallelic in *lcc*2 (Table 1). Of the two alleles of *lcc*1, one matched the HRE consensus sequence in 13 of the 15 nucleotides in U1 (Morin *et al*., 2012) and in 12 of 15 in SB65 (present work), and the other allele was identical for 10/15 nucleotides (Smith *et al*., 1998). The HRE in *lcc*2 matched the consensus sequence in 14 positions. There is presently no conclusive information correlating promoter strength with variation in the HRE sequence. Nevertheless, a reasonable speculation is that laccase gene induction by the toxin is regulated through the HRE and that *lcc*2 is induced as a consequence of the presence of two functional HRE copies that are lacking in *lcc*1. The relative roles of STRE and HRE and their combined effect on laccase expression in response to the stress of xenobiotic exposure should be studied further.

The higher basal transcript abundance and faster induction of the *lcc*2 gene in SB65 compared with U1 cannot be explained by promoter sequences with only one apparently insignificant nucleotide difference. It is likely that the variation between the two strains results from some, yet to be identified, upstream regulatory control in the pathway responsible for laccase production. Whatever mechanism is ultimately found to be important, the early response of SB65 may indicate a better toxin sensing ability, faster degradation of toxicity and increased resistance to green mould disease as compared with U1.

The introduction of the siRNA construct into *A. bisporus* gave very variable results that ranged from increased transcript abundance for both *lcc*1 and *lcc*2 to efficient knockdown of both genes. Significant variation in transcript abundances, including increases in some transformants, in response to siRNA treatment of *A. bisporus* was also seen by Eastwood and colleagues (2008) and Costa and colleagues (2009). One of the sources of the variation is likely to be changes in the nuclear status of the hypha where the number of nuclei per cell can vary from two to twelve (Molitoris *et al*., 1996). Unfortunately, the multinucleate condition also prevents the use of gene disruption as a tool for probing gene function. Inhibition of gene expression through siRNA has been clearly established for numerous organisms in an evolutionary conserved mechanism. The mechanism of upregulation of gene expression through siRNA, otherwise known as RNA activation (RNAa) is, however, poorly understood. The apparent activation observed here differs from RNAa in other systems in that the laccase siRNA construct targeted neither promoter regions nor gene-specific sense RNAs that regulate translation (Place *et al*., 2010) and that some transformants with the same construct showed increased transcript abundance and others showed decreased abundance. The reason for upregulation of the target genes, therefore, remains unclear. Nonetheless, overexpression was just as useful as knockdown in providing support for a role of laccases in resistance to the *T. aggressivum* toxin. Transformants that overexpressed the laccase genes were more resistant to the toxin than controls, and transformant W1 with a very low number of laccase transcripts was more sensitive. Transformants with laccase expression similar to the controls had toxin sensitivity that was similar to the controls. This inverse correlation of laccase transcript abundance with the sensitivity of the *A. bisporus* to *T. aggressivum* extract suggested that laccase activity is important in toxin metabolism.

Expression of *lcc*2 (Fig. 4B) corresponded well with laccase activity (Fig. 2) with and without toxin induction, and with toxin sensitivity of the transformants (Fig. 5A and B). No similar correspondence for *lcc*1 was apparent. These observations suggested that laccase 2 but not laccase 1 is an important contributor to *T. aggressivum* toxin resistance and likely to green mould disease resistance. It is also possible that other laccases play similar roles. Syringaldazine is a substrate that does not distinguish between the different laccase isoforms, and therefore laccase activity displayed in Figure 3 should represent the combined activity of all isoforms implicated by the genomic studies of Morin and colleagues (2012). Given that laccase activity was non-specific for the different laccase isoforms, it is conceivable that laccase 12, the primary isoform detected on semi-solid rye bran supplemented liquid (Hilden *et al*., 2013) or the expressed hypothetical laccase protein (JGI ID#209229), with a transcript induced by growth on compost (Morin *et al*., 2012), the only other laccases expressed to a significant extent, contribute to resistance and expression would mimic *lcc*2. Further studies on these genes and the relationship to green mould disease are warranted.

Green mould disease is one of the most devastating diseases to plague mushroom growers (Rinker, 1993; Anderson *et al*., 2001). Currently, the primary approach to managing a *T. aggressivum* problem is sanitation and hygiene (Rinker *et al*., 1997). Benzimidazole fungicides have also been used successfully for the treatment of green mould disease but isolates of *T. aggressivum* have already developed resistance (Romaine *et al*., 2005), and future outbreaks are possible. Based on the results in the present work, selection of strains of *A. bisporus* with increased laccase production or stimulation of laccase activities through addition of phenolic inducers to mushroom compost, shown to be an effective strategy for *L. edodes* production (Savoie *et al*., 2000), may offer alternate routes to disease reduction. Future work could include the identification of specific laccase gene inducers that might be used as disease control agents.

## Experimental procedures

### Organisms

*Agaricus bisporus* commercial strains Horst U1 off-white and large brown Sylvan SB65 (Sylvan America) were cultured on Complete Yeast Medium (CYM): 2% agar, 2% dextrose, 0.2% yeast extract, 0.2% peptone, 0.1% K_2_HPO_4_, 0.05% MgSO_4_·7H_2_O, 0.046% KH_2_PO_4_ (Raper *et al*., 1972). The cultures were maintained with minimal light exposure in a 28°C incubator. *Trichoderma aggressivum* f. *aggressivum* isolate 586 (Krupke *et al*., 2003) was grown on malt extract agar: 2% malt extract, 2% agar and incubated at 28°C.

After fungal growth, the plates were stored at 4°C. For long-term storage, samples of both species were archived in sterile 15% glycerol in cryogenic vials in liquid nitrogen. Active cultures were established periodically for further use.

### *Trichoderma aggressivum* toxin inhibition of *A. bisporus*

Conidia from a mature *T. aggressivum* culture were suspended in water and used to inoculate 500 ml of 2% malt extract broth (Difco) to yield a final concentration of 10^5^ conidia ml^−1^. The liquid culture was incubated at room temperature with shaking at 120 r.p.m. and 12 h of light per day for 30 days (Krupke *et al*., 2003). After growth, the mycelium was removed by filtration through Fisherbrand Filter Paper P8. Organic components of the spent medium were collected by liquid–liquid extraction with dichloromethane (DCM).

To confirm toxicity, the extract was pipetted in 10 μl aliquots onto a 5 mm disc of Whatman #1 filter paper. We had previously determined that DCM inhibits the growth of *A. bisporus* and so the solvent was evaporated for 5 min before the next aliquot was applied and prior to inhibition testing. The filter paper with the extract was added to a lawn of *A. bisporus* and incubated at 28°C. The lawn was prepared by removing mycelium from an agar plate, homogenizing the colony in liquid CYM using a Sorvall omni mixer for 30 s at high speed, and inoculating the homogenate onto CYM plates. Inhibition zones around the filter paper were measured to quantify toxicity of the crude extract. In experiments using the toxin to challenge *A. bisporus* in broth culture, DCM was completely removed by rotary evaporation and the dried extract was suspended in 50% methanol. Liquid cultures of *A. bisporus* were prepared by homogenizing mycelia as above. A microtiter plate growth inhibition assay was prepared by adding 200 μl of homogenized mycelia to each well, followed by treatment with *T. aggressivum* toxin, or solvent, ranging from 0.2 μl (0.1%) to 5 μl (2.5%). *Agaricus bisporus* growth was measured by absorbance at 595 nm every 24 h for 7 days.

### Detoxification using exogenous laccase

To determine if laccase could alter the toxicity of the *T. aggressivum* extract, 4 U of commercially available *A. bisporus* laccase (Sigma-Aldrich, SKU# 40452) and varying volumes of the extract were incubated overnight at 21°C. Because the metabolites in the *T. aggressivum* extract were only slightly soluble in water, we confirmed that laccase was active against syringaldazine (Sigma-Aldrich, SKU # 177539) in 50% methanol, and this solvent was used in the detoxification tests. The overnight reaction was added to a microtiter plate that contained 200 μl of homogenized *A. bisporus* mycelium to final concentrations of 0.25%, 0.5%, 0.75% v/v. Growth was recorded as absorbance at 595 nm every 24 h for 10 days.

### Measurement of total laccase activity

Liquid still cultures of *Agaricus* were prepared by homogenizing mycelia as above and pouring the homogenate into empty sterile Petri plates. The plates were incubated at room temperature for 20 days to increase biomass, followed by treatment with 100 μl (0.5%) of *T. aggressivum* extract per 20 ml culture. Control cultures were treated with 100 μl 50% methanol. Intracellular protein was isolated as described by Criquet and colleagues (1999) at day 0, 2 and 4. The mycelium was frozen and crushed in liquid nitrogen and 1 g suspended in 10 ml of 0.1 M CaCl_2_. The mixture was shaken (1 h, 120 r.p.m., 21°C), filtered (Whatman #4 filter paper), centrifuged (20 min, 12 000 × *g*, 4°C), and the supernatant collected and filtered through 8 μm filter paper (Millipore). The protein solution was dialysed (12–14 kDa cut-off, Fisher Scientific) overnight at 4°C against 2 mM bis-Tris, dried over polyethylene glycol and resuspended into 1 ml of 2.5 mM CuSO_4_. Protein concentration was measured with the Bradford (1976) assay.

Laccase activity was determined by the oxidation of syringaldazine where one unit of laccase enzyme is defined as the change in absorbance at 525 nm of 0.001 per minute using 1 μg of total protein (Ride, 1980). Each reaction included 3 ml of phosphate buffer (pH 6), 100 μl of 2.5 mM syringaldazine (in 90% ethanol, 5% methanol, 5% isopropanol) and 5 μg of total protein. The reaction was allowed to proceed for 60 s before reading absorption at 525 nm.

### Cloning and sequencing of *lcc*1 and *lcc*2

Entire sequences for *lcc*1 and *lcc*2 were obtained from both U1 and SB65 using PCR with multiple primers based upon the sequence (GenBank Accession No. L10664.1) from *A. bisporus* strain D469 to generate several clones. The PCR fragments were ligated into pUC19 linearized with HincII (New England Biolabs) and transformed into *Escherichia coli* DH5α (Invitrogen). Each insert was sequenced and the information was assembled and aligned with Vector NTI Advance 11 and Gene Doc 2.7.0 (Invitrogen) to generate composite 6572 bp sequences for both *Agaricus* strains. The genes are found in tandem within the genome (Smith *et al*., 1998) and the sequenced region started at −422 bp of the *lcc*1 start codon and ended at the translation stop codon of *lcc*2. The assembly of these sequences permitted the development of real-time PCR measurements of transcript abundances of the two genes as given below. The information also included *lcc*1 and *lcc*2 promoter regions that were examined for regulatory motifs that might explain differences in regulation of the genes in the two strains.

### Measurement of *lcc*1 and *lcc*2 transcript abundance

*Agaricus bisporus* mycelium was harvested from liquid cultures treated with *T. aggressivum* extract or non-treated controls by filtration through Whatman #4 filter paper, frozen in liquid nitrogen and crushed with mortar and pestle. Extraction of total RNA was performed using the Norgen Biotek Total RNA Purification Kit. Genomic DNA was removed from the RNA samples by digestion with DNase I (Ambion). The concentration and purity of RNA was measured on a NanoVue Plus Spectrophotometer and stored at −80°C.

Superscript III First-Strand Synthesis System for reverse transcriptase polymerase chain reaction (RT-PCR) (Invitrogen) was used to generate complementary DNA (cDNA). The cDNA was used as a template in quantitative PCR (qPCR). qPCR was performed using the Bio-Rad iCycler iQ Real-Time PCR Detection System with 96 well plates and the Bio-Rad iQ SYBR Green Supermix. Following 95°C for 5 min enzyme activation, 40 cycles of 95°C for 15 s and 58°C for 30 s were used for amplification and fluorogenic responses recorded during each elongation step. The threshold cycle (C_T_) was determined by machine software. Specific primers were designed for *lcc*1, *lcc*2 and *β-tub* (Table S1). The laccase primers had no significant homology with any of the other laccase genes or pseudogenes identified by Morin and colleagues (2012). Lack of amplification from an *lcc*1 template with *lcc*2 primers or from an *lcc*2 template with *lcc*1 primers confirmed specificity.

Plasmids containing partial copies of *lcc*1, *lcc*2 and *β-tub* were created to allow estimation of the number of cDNA copies in an experimental sample with a modification of the procedures of Hou and colleagues (2010). Clones were generated using PCR with *Taq* polymerase (Fermentas). PCR fragments were ligated into pDRIVE (Qiagen) and cloned into *E. coli* DH5α. Following extraction, plasmid concentrations were measured by spectroscopy. The mass of a single plasmid was calculated from the DNA sequence and was used to determine the number of plasmid copies in a sample. A dilution series of the samples was used in qPCR to generate standard curves of critical value (C_T_) versus calculated copy number for *lcc*1, *lcc*2 and *β-tub*. The C_T_ of each experimental sample in qPCR was compared with the standard curve to determine number of copies of each cDNA. The copy number of *β-tub* was normalized to 1000 cDNAs, and copy numbers of *lcc*1 and *lcc*2 cDNAs were determined relative to that standard.

### Laccase siRNA

The laccase RNA silencing construct was cloned into a derivative of plasmid pFGC1008 that was obtained from the *Arabidopsis* Information Resource. The vector was modified by the replacement of the 35S promoter with the glyceraldehyde 3-phosphate dehydrogenase promoter from *A. bisporus* (Abubaker, 2010). The siRNA construct was based on a region that is homologous in both *lcc*1 and *lcc*2 in both U1 and SB65 and in a region distant from the real-time PCR primer sites. The amplicon of 467 bp from cDNA included restriction enzyme sites to allow orientation when cloning. The laccase sense segment was cloned into the Asc1 and Swa1 sites of the plasmid, and the antisense segment was cloned into the BamH1 and Spe1 sites. The two segments flanked a 372 bp *GUS* spacer. Plasmid construction was verified by restriction enzyme digestion, PCR and sequence analyses.

The engineered plasmid was introduced into *A. tumefaciens* strain AGL-1 by electroporation (Mattanovich *et al*., 1989), and transformants were selected on Luria-Bertani (LB) supplemented with 100 μg ml^−1^ carbenicillin and 50 μg ml^−1^ chloramphenicol. *Agaricus bisporus* gill tissue was transformed according to the methods developed by Chen and colleagues (2000). Potential transformants were selected for 21 days on CYM plates with 25 μg ml^−1^ hygromycin, followed by growth for another 21 days on CYM with 50 μg ml^−1^ hygromycin. Colonies were transferred to non-selective CYM plates and the presence of the siRNA construct was confirmed by PCR. All transformants were tested for toxin sensitivity and *lcc*1 and *lcc*2 transcript abundance after a 4-day incubation with *T. aggressivum* extract.

### Data and statistical analysis

All data were assimilated into Microsoft Excel 2010 to generate the figures. The figures and pictures were transferred to Photoshop CS6 for formatting and assembly.

All the statistical analysis was performed using IBM® SPSS® Statistics version 20. An ANOVA test was used to compare samples within one time point, while ANOVA with repeated measures was used to measure significant difference between conditions over time.

## Conflict of interest

None declared.

## Supporting Information

**Table S1.** Primers used in this study.

## References

[b1] Abubaker KS (2010) Cell wall degrading enzymes and in*Trichoderma aggressivum and teractions Agaricus bisporus*. PhD Thesis. St. Catharines, ON, Canada: Brock University

[b2] Abubaker KS, Sjaarda C, Castle AJ (2013). Regulation of three genes encoding cell wall degrading enzymes of *Trichoderma aggressivum* during interaction with *Agaricus bisporus*. Can J Microbiol.

[b3] Anderson MG, Beyer DM, Wuest PJ (2001). Yield comparison of hybrid *Agaricus* mushroom strains as a measure of resistance to *Trichoderma* green mold. Plant Dis.

[b4] Baldrian P (2004). Increase of laccase activity during interspecific interactions of white-rot fungi. FEMS Microbiol Ecol.

[b5] Baldrian P (2006). Fungal laccases – occurrence and properties. FEMS Microbiol Rev.

[b6] Bradford MM (1976). A rapid and sensitive method for the quantification of microgram quantities of protein utilizing the principle of protein-dye binding. Anal Biochem.

[b7] Chen X, Stone M, Schlagnhaufer C, Romaine CP (2000). A fruiting body tissue method for efficient *Agrobacterium-*mediated transformation of *Agaricus bisporus*. Appl Environ Microbiol.

[b8] Chet I, Harman GE, Baker R (1981). *Trichoderma hamatum*: its hyphal interactions with *Rhizoctonia solani* and *Pythium* spp. Microb Ecol.

[b9] Cooney JM, Lauren DR (1999). Biotransformation of the *Trichoderma* metabolite, 6-n-pentyl-2H-pyran-2-one (6PAP) by selected fungal isolates. J Nat Prod.

[b10] Cooney JM, Lauren DR, di Menna ME (2001). Impact of competitive fungi on trichothecene production by *Fusarium graminearum*. J Agric Food Chem.

[b11] Costa ASMB, Thomas DJI, Eastwood D, Cutler SB, Baily AM, Foster GD (2009). Quantifiable down regulation of endogenous genes in *Agaricus bisporus* mediated by expression of RNA hairpins. J Microbiol Biotechnol.

[b12] Criquet S, Tagger S, Vogt G, Iacazio G, Le Petit J (1999). Laccase activity of forest litter. Soil Biol Biochem.

[b13] Eastwood DC, Challen MP, Zhang C, Jenkins H, Henderson J, Burton KS (2008). Hairpin-mediated down-regulation of the urea cycle enzyme argininosuccinate lyase in *Agaricus bisporus*. Mycol Res.

[b14] Eastwood DC, Bains NK, Henderson J, Burton KS (2011). Genome organization and transcription response to harvest of two metallothionein-like genes in *Agaricus bisporus* fruiting bodies. J Microbiol Biotechnol.

[b15] Geels FP (1997). Rondetafel-bijeenkomst over *Trichoderma* [Roundtable meeting over *Trichoderma*]. Champignoncultuur.

[b16] Guthrie JL, Castle AJ (2006). Chitinase production during interaction of *Trichoderma aggressivum* and *Agaricus bisporus*. Can J Microbiol.

[b17] Hilden K, Makela MR, Lankinen P, Lundell T (2013). *Agaricus bisporus* and related *Agaricus* species on lignocellulose: production of manganese peroxidase and multicopper oxidases. Fungal Genet Biol.

[b18] Hou Y, Zhang H, Miranda L, Lin S (2010). Serious overestimation in quantitative PCR by circular (supercoiled) plasmid standard: microalgal *pcna* as the model gene. PLoS ONE.

[b19] Janusz G, Kucharzyk KH, Pawlik A, Staszczak M, Paszczynski AJ (2013). Fungal laccase, manganese peroxidase and lignin peroxidase: gene expression and regulation. Enzyme Microb Technol.

[b20] Juo ZS, Chiu TK, Leiberman PM, Baikalov I, Berk AJ, Dickerson RE (1996). How proteins recognize the TATA box. J Mol Biol.

[b21] Kobayashi N, McEntee K (1993). Identification of cis and trans components of a novel heat shock stress regulatory pathway in *Saccharomyces cerevisiae*. Mol Cell Biol.

[b22] Krupke OA, Castle AJ, Rinker DL (2003). The North American mushroom competitor, *Trichoderma aggressivum* f. *aggressivum*, produces antifungal compounds in mushroom compost that inhibit mycelial growth of the commercial mushroom *Agaricus bisporus*. Mycol Res.

[b23] Largeteau ML, Savoie J-M (2010). Microbially induced diseases of *Agaricus bisporus*: biochemical mechanisms and impact on commercial mushroom production. Appl Microbiol Biotechnol.

[b24] Larrondo LF, Avila M, Salas L, Cullen D, Vicuna R (2003). Heterologous expression of laccase cDNA from *Ceriporiopsis subvermispora* yields copper-activated apoprotein and complex isoform patterns. Microbiology.

[b25] Leonowicz A, Cho NS, Luterek J, Wilkolazka A, Wojtas-Wasilewska M, Matuszewska A (2001). Fungal laccase: properties and activity on lignin. J Basic Microbiol.

[b26] Mamoun ML, Iapicco R, Savoie JM, Van Griensven LJLD, Olivier JM (2000). Green mould disease in France: *Trichoderma harzianum* Th2 and other species causing damages on mushroom farms. Science and Cultivation of Edible Fungi.

[b27] Mattanovich D, Rüker F, Machado AC, Laimer M, Regner F, Steinkellnew H (1989). Efficient transformation of *Agrobacterium* spp. by electroporation. Nucleic Acids Res.

[b28] Mayer AM, Staples RC (2002). Laccase: new functions for an old enzyme. Phytochemistry.

[b29] Molitoris PH, Buchalo AS, Wasser SP, Grigansky AP (1996). Studies of the vegetative mycelium in the genus *Agaricus* L.: Fr. emend. Karst. Botany and Mycology for the Next Millennium.

[b30] Morin E, Kohler A, Baker AR, Foulongne-Oriol M, Lombard V, Nagy LG (2012). Genome sequence of the button mushroom *Agaricus bisporus* reveals mechanisms governing adaptation to a humic-rich ecological niche. Proc Natl Acad Sci USA.

[b31] Nagai M, Kawata M, Watanabe H, Ogawa M, Saito K, Takesawa T (2003). Important role of fungal intracellular laccase for melanin synthesis: purification and characterization of an intracellular laccase from *Lentinula edodes* fruit bodies. Microbiology.

[b32] Perry CR, Smith M, Britnell CH, Wood DA, Thurston CF (1993). Identification of two laccase genes in the commercial mushroom *Agaricus bisporus*. J Gen Microbiol.

[b33] Piscitelli A, Giardina P, Lettera V, Pezzella C, Sannia G, Faraco V (2011). Induction and transcriptional regulation of laccases in fungi. Curr Genomics.

[b34] Place RF, Noonan EJ, Földes-Papp Z, Li LC (2010). Defining features and exploring chemical modifications to manipulate RNAa activity. Curr Pharm Biotechnol.

[b35] Poole PR, Whitaker G (1997). Biotransformation of 6-pentyl-2-pyrone by *Botrytis cinerea* in liquid cultures. J Agric Food Chem.

[b36] Raper CA, Raper JR, Miller RE (1972). Genetic analysis of the life cycle of *Agaricus bisporus*. Mycologia.

[b37] Reino JL, Guerrero RF, Hernández-Galan R, Collado IG (2008). Secondary metabolites from the biocontrol agent *Trichoderma*. Phytochem Rev.

[b38] Ride JP (1980). The effect of induced lignification on the resistance of wheat cell walls to fungal degradation. Physiol Plant Pathol.

[b39] Rinker DL (1993). Disease management strategies for *Trichoderma* mould. Mushroom World.

[b40] Rinker DL, Alm G (1997). Investigations of factors influencing the expression of green mould. Mushroom World.

[b41] Rinker DL, Alm G, Castle AJ, Rghei N (1997). Distribution of green mould project on infected mushroom farms. Mushroom World.

[b42] Romaine CP, Royse DJ, Schlagnhaufer B (2005). Superpathogenic *Trichoderma* resistant to TopsinM found in Pennsylvania and Delaware. Mushroom News.

[b43] Savoie J-M, Mata G (2003). *Trichoderma harzianum* metabolites pre-adapt mushrooms to *Trichoderma aggressivum* antagonism. Mycologia.

[b44] Savoie J-M, Mata G, Billette C (1998). Extracellular laccase production during hyphal interactions between *Trichoderma* sp. and Shiitake, *Lentinula edodes*. Appl Microbiol Biotechnol.

[b45] Savoie J-M, Delpech P, Billette C, Van Griensven LJLD, Mata G (2000). Inoculum adaptation changes the outcome of the competition between *Lentinula edodes* and *Trichoderma* spp. during Shiitake cultivation on pasteurized wheat straw. Science and Cultivation of Edible Fungi.

[b46] Savoie J-M, Mata G, Mamoun M (2001). Variability in brown line formation and extracellular laccase production during interaction between white-rot basidiomycetes and *Trichoderma harzianum* biotype Th2. Mycologia.

[b47] Score AJ, Palfreyman JW, White NA (1997). Extracellular phenoloxidase and peroxidase enzyme production during interspecific fungal interactions. Int Biodeterior Biodegradation.

[b48] Seaby D (1987). Infection of mushroom compost by *Trichoderma* species. Mushroom J.

[b49] Smith M, Shnyreva A, Wood DA, Thurston CF (1998). Tandem organization and highly disparate expression of the two laccase genes *lcc*1 and *lcc*2 in the cultivated mushroom *Agaricus bisporus*. Microbiology.

[b50] Sobieralski K, Siwulski M, Frużyńska-Jóźwiak D, Górski R (2009). Impact of *Trichoderma aggressivum* f. *europaeum* Th2 on the yielding of *Agaricus bisporus*. Phytopathologia.

[b51] Sorger PK (1991). Heat shock factor and the heat shock response. Cell.

[b52] Thurston CF (1994). The structure and function of fungal laccases. Microbiology.

[b53] Treger JM, Magee TR, McEntee K (1998). Functional analysis of the stress response element and its role in the multistress response of *Saccharomyces cerevisiae*. Biochem Biophys Res Commun.

[b54] Trejo-Hernandez MR, Lopez-Munguia A, Ramirez RQ (2001). Residual compost of *Agaricus bisporus* as a source of crude laccase for enzymatic oxidation of phenolic compounds. Process Biochem.

[b55] Velázquez-Cedeño M, Farnet AM, Billette C, Mata G, Savoie J-M (2007). Interspecific interactions with *Trichoderma longibrachiatum* induce *Pleurotus ostreatus* defence reactions based on the production of laccase isozymes. Biotechnol Lett.

[b56] Yang HH, Yang SL, Peng KC, Lo CT, Liu SY (2009). Induced proteome of *Trichoderma harzianum* by *Botrytis cinerea*. Mycol Res.

