# Supplementary material for: Induction of lcc2 expression and activity by Agaricus bisporus provides defence against Trichoderma aggressivum toxic extracts
Source: Microb Biotechnol. 2015 Mar 30;8(6):918–29. doi: 10.1111/1751-7915.12277 (PMC4621445; doi:10.1111/1751-7915.12277)
Supplement: Supplementary file 1 — Table S1. Primers used in this study. [file mbt20008-0918-sd1.docx]

Supporting Information

Table1. Primers used in this study.

| Primer name | Sequence (5’-3’) | Target | Use |
| --- | --- | --- | --- |
| AL1F-RT | CCACGTGATCCACTCAG | *lcc*1 | Realtime PCR |
| AL1R-RT | TGAAAAACTCGTTAGTGGC |  |  |
| AL2F-RT | CTGAGGATCCACTCGGG | *lcc*2 | Realtime PCR |
| AL2R-RT | AGCTGAAGAATTCGTTGTTGA |  |  |
| Β-tub 1 | TTTCGCCTCAAACCCTCG | *β-tub* | Realtime PCR |
| Β-tub 2 | TCCCAGAACTTGGCACCA |  |  |
| Lcc RNAiF | TCAGGCGCGCCACTAGTCGATAAGCACATCACCCTCAAC | *lcc*1 and *lcc*2 | SiRNA |
| Lcc RNAiR | CTCATTTAAATGGATCCCTTGAGGAGTGATCTGAGACTGG |  |  |
